# MiR-423 is differentially expressed in patients with stable and unstable coronary artery disease: A pilot study

**DOI:** 10.1371/journal.pone.0216363

**Published:** 2019-05-06

**Authors:** Barbara Rizzacasa, Elena Morini, Ruggiero Mango, Chiara Vancheri, Simone Budassi, Gianluca Massaro, Sara Maletta, Massimiliano Macrini, Silvio D’Annibale, Francesco Romeo, Giuseppe Novelli, Francesca Amati

**Affiliations:** 1 Department of Biomedicine and Prevention, University of Rome Tor Vergata, Rome, Italy; 2 Complex Operative Unit of Cardiology, Policlinico Tor Vergata- PTV Foundation, Rome, Italy; 3 Department of System Medicine, University of Rome Tor Vergata, Rome, Italy; 4 Complex Operative Unit of Medical Genetics, Policlinico Tor Vergata- PTV Foundation, Rome, Italy; 5 Department of Human Sciences and Quality of Life Promotion, University San Raffaele, Rome, Italy; University of Cincinnati College of Medicine, UNITED STATES

## Abstract

Coronary artery disease (CAD) and acute myocardial infarction (AMI) are the leading causes of death worldwide. Since only a subset of CAD patients develops myocardial infarction, it is likely that unique factors predispose to AMI. Circulating microRNAs represent diagnostic powerful biomarkers for detection of heart injuries and patients’ risk stratification. Using an array-based approach, the expression of 84 circulating miRNAs was analyzed in plasma of pooled stable CAD patients (CAD; n = 5) and unstable CAD patients (AMI_T0; n = 5) enrolled within 24 hours from an AMI event. The array experiments showed 27 miRNAs differentially expressed with a two-fold up- or down-regulation (10 up- and 17 down-regulated miRNAs). Among them, miR-423-5p dis-regulation was confirmed in a larger case study (n = 99). Circulating miR-423-5p resulted to be significantly down-regulated within 24 hours from the AMI event (FC = -2, p≤0.05). Interestingly, miR-423-5p expression resulted to be increased (FC = +2; p≤0.005) in a subgroup of the same AMI patients (AMI_T1; n = 11) analyzed after 6 months from the acute event. We extended miR-423-5p expression study on PBMCs (peripheral blood mononuclear cells), confirming also in this tissue its up-regulation at 6 months post-AMI. Receiver operating characteristic analyses (ROC) were performed to detect the power of miR-423-5p to discriminate stable and unstable CAD. In plasma, miR-423-5p expression accurately distinguishes stable and unstable CAD patients (AUC = 0.7143, p≤0.005). Interestingly, the highest discriminatory value (AUC = 0.8529 p≤0.0005) was identified in blood cells, where miR-423-5p expression is able to differentiate unstable CAD patients during an acute event (AMI_T0) from those at six months post-AMI (AMI_T1). Furthermore, cellular miR-423-5p may discriminate also stable CAD patients from unstable CAD patients after six months post-AMI (AUC = 0.7355 p≤0.05). The results of this pilot-study suggest that miR-423-5p expression level both in plasma and blood cells, could represent a new promising biomarker for risk stratification of CAD patients.

## Introduction

Coronary artery disease (CAD) refers to the progressive development of atherosclerotic plaques in blood vessels and represents one of the leading causes of morbidity and mortality worldwide. Atherosclerosis is a complex process that begins with endothelial cells dysfunction in the coronary arteries and can ultimately lead to a narrowing of the vessels obstructing the blood flow to the heart and causing acute myocardial infarction (AMI) [1–3]. The evidence that only a subgroup of patients with CAD will develop myocardial infarction suggests that unique features can predispose to AMI. Even if numerous studies investigated the pathogenesis of atherosclerosis and the development of ischemic heart disease, the mechanisms that regulate plaque stability, thus determining which subgroup of patients is more prone to develop acute coronary syndrome, are not yet completely understood [4,5].

Epigenetic mechanisms emerged as important players in the development of pathological cardiovascular phenotypes, such as coronary atherosclerosis. Importantly, epigenetic modifications represent not only a key for a better understanding of the complex background of cardiovascular diseases but also a new field of investigation for the discovery of new biomarkers with diagnostic and prognostic purposes [6].

Among the best studied epigenetic mechanisms, microRNAs (miRNAs) regulate gene expression at post-transcriptional level by binding specific mRNA targets and are critically involved in important biological processes in healthy and diseased conditions, including cardiovascular diseases [7,8]. Recently, miRNAs have been described as key regulators of important pathways, such as cellular adhesion, proliferation, and inflammation, which are central for atherosclerosis development [9]. In addition, miRNAs can be detected in serum or plasma with a remarkable stability [10]. Circulating miRNAs not only present many of the essential features of good biomarkers but they could also play a fundamental role in risk prediction for AMI and patients’ stratification with the final goal of personalized medicine [11]. Previous case-control studies identified a high number of miRNAs differentially expressed in plasma of CAD and AMI patients, such as miR-1, -122, -126, -133a/b, -199a [12], miR-499, and miR-208a [13], as a result of cardiomyocyte necrosis and their consequential release into the bloodstream [11,14–16].

The vast majority of data literature on the identification of epigenetic biomarkers are based on the study of patients affected by coronary artery disease in comparison with healthy control subjects. Aim of this study is the identification of epigenetic biomarkers useful for the discrimination of patients with stable and unstable CAD, so we focused our analysis only on subjects already affected by coronary atherosclerosis. Moreover, since epigenetic patterns can quickly change over time and under different environmental stimuli and conditions, we analyzed patients with stable and unstable CAD at the time of hospitalization (T0, CAD and AMI_T0 groups) and a subgroup of AMI patients, who accepted to participate to the study, at 6 months post-AMI (T1, AMI_T1 group).

In this pilot-study, using a Real Time-PCR Array-based method, we analyzed the expression of 84 miRNAs commonly expressed in plasma. Our analysis revealed that miR-423-5p is differentially expressed in plasma and PBMCs of patients with stable and unstable coronary artery disease. ROC curve analyses showed that plasma miR-423-5p level has a good discriminatory ability to separate stable CAD patients from unstable CAD patients during the first 24 hours of onset of acute myocardial infarction symptoms (AMI_T0). Moreover, in PBMCs, miR-423-5p expression is able to discriminate unstable CAD patients at 6 months post-AMI (AMI_T1) from both AMI_T0 and CAD patients.

Our findings make the way to the use of miR-423-5p as potential epi-biomarker for the identification of patients with unstable CAD and highlight the importance of deeper studies on the expression of this miRNA in order to understand its functional role in coronary artery disease development.

## Materials and methods

### Participants’ recruitment and samples collection

We enrolled 99 patients (from January 2017 to September 2017), including 61 patients with chronic stable coronary artery disease (CAD group) and 38 patients arrived at the attention of the Unit of Cardiology (Policlinico Tor Vergata, Rome) during a myocardial infarction event (AMI group). One blood sample (10 mL) has been collected during the hospitalization and within 24 hours from the onset of myocardial event (AMI_T0 group) or within 24 hours from the coronary angiography (CAD group). For both patients groups each blood sample has been obtained after percutaneous coronary intervention (PCI); for those patients with AMI who accepted to participate, another blood sample was achieved at 6 months post-AMI (AMI_T1; n = 11), dx.doi.org/10.17504/protocols.io.zpvf5n6 [PROTOCOL DOI].

CAD group includes patients with known or suspected coronary artery disease, admitted to the hospital for provocative cardiac testing positive for inducible myocardial ischemia. Only patients with angiographically documented CAD, were enrolled in this study. AMI group includes patients arrived at the emergency department with signs or symptoms of acute myocardial ischemia and diagnosed with ST segment elevation (STEMI n = 23) or non-ST segment elevation (NSTEMI = 15) acute myocardial infarction according to the definition of International guidelines [4].

Patients less than 50-year-old, or with heart failure, neoplastic disease, autoimmune disease, inflammatory chronic disease, chronic kidney disease (creatinine clearance <15 ml/min) and previous events of acute myocardial infarction have been excluded from this study.

All the principles outlined in the Helsinki Declaration of 1975, as revised in 2013 [17], have been followed in all the experiments involving human subjects during the current study. All patients received and signed a written informed consent. The Ethical Committee of Policlinico Tor Vergata (Rome) approved this project (n. 30/15).

### Plasma and PBMCs isolation

Samples of plasma and PBMCs have been isolated by whole blood using Ficoll Plaque Plus (GE Healthcare, Little Chalfont, UK) according to manufacturer instructions. Isolated PBMCs have been suspended in 1mL of Trizol (Ambion, Waltham, MA, USA) and stored at -80°C until further analysis. Plasma samples were centrifuged for 10’ at 16,000 x g in order to remove additional nucleic acids attached to cell debris and then stored at -80°C until further analysis.

### Total RNA extraction from plasma samples and reverse transcription

Total RNA, including microRNAs, was extracted from 100μl of plasma using miRNeasy Serum/Plasma Kit (QIAGEN) according to the manufacturer’s instructions. To monitor miRNAs’ isolation, a spike-in control (*Caenorhabditis Elegans* Ce_miR-39_1, miRNeasy Serum/Plasma Spike-In Control, QIAGEN) was used at the time of the extraction. Subsequently, a specific miScript primer assay for *Caenorhabditis Elegans* Ce_miR-39_1 (QIAGEN) was used to assess the quality of RNA extraction by quantitative Real Time PCR (qRT-PCR). Based on this evaluation, 1.5μl of total RNA has been reverse transcribed into cDNA using the miScript II RT kit (QIAGEN) following the manufacturer’s instruction.

### Total RNA extraction from PBMCs and reverse transcription

RNA extraction from PBMCs was performed using Trizol reagent according to manufacturer instructions. RNA concentration was evaluated by using a NanoDrop ND-1000 Spectrophotometer (Euro-Clone). To isolate miRNA fraction, 100ng of RNA has been reverse transcribed using the miScript II RT kit (QIAGEN) following the manufacturer’s instruction [18].

### Human Serum & Plasma miScript miRNA PCR Array

Human Serum & Plasma miScript miRNA PCR Array (MIHS-106ZA, QIAGEN) is a pre-set array that enables the rapid profiling of the 84 most relevant circulating miRNAs associated with serum and plasma.

We profiled the expression of these 84 circulating miRNAs in pooled plasma RNA samples obtained from CAD (n = 5) and AMI_T0 (n = 5) patients, matched for age and clinical characteristics according to their medical condition (S1 Table). Amplification reactions were performed using the Human Serum & Plasma miScript miRNA PCR Array with miScript SYBR Green PCR kit (QIAGEN) in a 7300 quantitative Real Time PCR (qRT-PCR) system (Applied Biosystem) following the manufacturer’s instruction. Three independent array experiments have been conducted for each pool (n = 3). A web-based data analysis tool (GeneGlobe Data Analysis Center, www.qiagen.com) was used for statistical analysis of PCR Array data. This online software uses threshold cycle (Ct) values to calculated miRNA expression in each category of samples, so accordingly, miRNAs expression is classified as high (Ct <25), good (Ct range 25–30), low (Ct range 30–35) and undetectable (Ct >35).

Since for the expression study of circulating miRNAs there is not a current consensus on the use of specific house-keeping miRNAs for data normalization, we applied a global normalization including the average threshold cycle (Ct) of all the 84 miRNAs in the array plates to calculate miRNAs’ expression according to manufacturer’s instructions (S2 Table) [19].

### miRNA-specific expression by qRT-PCR

The qRT-PCR expression analyses were performed on all the recruited patients (61 CAD, 38 AMI_T0 and 11 AMI_T1 patients respectively) in triplicate and for at least three independent experiments (n = 3) by using miScript SYBR Green PCR kit (QIAGEN) and miRNA-specific miScript Primer Assays (S1 File). As housekeeping miRNA for data normalization we selected miR-15b-5p dx.doi.org/10.17504/protocols.io.zpxf5pn [PROTOCOL DOI] and evaluated its expression on all our samples. Since we did not observe any significant differences in miR-15b-5p expression among CAD, AMI_T0 and AMI_T1 patients (S1 Fig), this microRNA was used for data normalization and analysis of the results. Data analysis was performed using the comparative Ct method quantification (2^-ΔCt^ method), dx.doi.org/10.17504/protocols.io.zp7f5rn [PROTOCOL DOI] [20].

### Statistics

For the statistical analyses of miRNAs’ expression, only miRNAs with a threshold cycle (Ct) value <35 were considered. Kolmogorov-Smirnov test was used to analyze the distribution of expression data from qRT-PCR assays. Student T test, Mann-Whitney test, and Kruskal-Wallis followed by Dunn’s test for multiple comparisons were used for data analysis as appropriate. For non-parametric distribution, expression data are represented as median and range; for parametric distribution, expression data are represented as mean and standard deviation. Clinical characteristic differences have been analyzed using Student T and data are represented as mean and standard deviation.

The Receiver Operating Characteristic (ROC) curve was used to determine the specificity of miR-423-5p to discriminate among patients’ groups. Significance was set at p≤0.05. Statistical analysis was performed using GraphPad Prism 6.0 (GraphPad Software, San Diego, CA, USA).

## Results and discussion

### Subjects

We enrolled stable CAD patients (CAD) and patients with acute myocardial infarction (AMI) starting from January 2017 to September 2017. All patients underwent coronary angiography in order to assess the presence of coronary artery disease and its degree, avoiding the risk of recruiting false positive patients. Primary PCI was the first procedure for treating the culprit lesion in AMI group; any other hemodynamically significant lesions were treated during the same procedure or in a staged coronary intervention. Patients in the CAD group were treated with angioplasty when hemodynamically significant lesions were found. The main clinical features of all patients are reported in Table 1.

**Table 1:** Clinical characteristics of CAD and AMI patients.

	CAD patients (n = 61)	AMI patients (n = 38)	p-value
**Age (years)**	66.5±9.5	64.5±11.9	n.s.
**Height (cm)**	165.5±21.9	169.3±8.4	n.s.
**Weight (kg)**	79.4±11.3	81.6±12.3	n.s.
**BMI (kg/m**^**2**^**)**	27.8±3.8	28.4±3.3	n.s.
**Hypertension (%)**	92	95	n.s.
** SBP (mmHg)**	131.3±16.8	138.8±24.6	n.s.
** DBP (mmHg)**	75.5±8.2	83.6±13.7	p≤0.0005
**Type 2 diabetes (%)**	44	24	p≤0.05
**Dyslipidemia (%)**	88	84	n.s.
**Smoking history**			
** Present (%)**	16	59	p≤0.0005
** Past (%)**	54	14	p≤0.0005
**EF (%)**	53.4±8.6	45.8±9.3	p≤0.0005
**LVDD (mm)**	47.3±6.5	47.8±4.7	n.s.
**LVDD/BSA (mm/m**^**2**^**)**	25.5±3.4	24.7±2.5	n.s.
**Number of affected vessels**			
** 1 vessel disease (%)**	40	43	n.s.
** 2 vessel disease (%)**	30	35	n.s.
** 3 vessel disease (%)**	30	22	n.s.
**Type of affected vessel (degree of stenosis >50%)**			
** LAD**^a^ **(%)**	79	70	n.s.
** CFX**^b^ **(%)**	60	41	n.s.
** RCA**^c^ **(%)**	53	62	n.s.
**Cardiac Troponin I (μg/L)**	-	49±68	

Continuous data are expressed as mean ± SD; categorical data are expressed as percentage. Student T test was used to assess significance.

^a^LAD, left descending artery.

^**b**^CFX, circumflex coronary artery.

^**c**^RCA, right coronary artery.

Statistically significant differences between CAD and AMI patients were observed for smoking history, type 2 diabetes mellitus, DBP and EF (Table 1). Smoking confirms to be an important risk factor in our patients’ group; 59% of AMI patients were smokers at the time of the event, compared to 16% of patients with stable CAD (p≤0.0005), while the percentage of ex-smoker was higher in CAD compared to AMI patients (54% and 14%, respectively, p≤0.0005). Twenty-seven CAD patients (44%) have type 2 diabetes mellitus respect to AMI patients (24%, p≤0.05). Arterial hypertension is highly represented in both groups; however, we noticed that the DBP values of patients with unstable CAD (AMI) tend to be higher compared to patients with stable CAD, probably because CAD patients were already under medical treatments in order to control hypertension. From the analysis of the echocardiographic parameters, it appears, as expected, that patients with unstable CAD (AMI) present a lower percentage of ejection fraction (EF) compared to patients with stable CAD (p≤0.0005). Finally, we analyzed the type of vessels affected by critical disease (i.e. vessels with a stenosis >50%) and found that the left anterior descending artery (LAD) was more frequently affected than circumflex artery (CFX) and right coronary artery (RCA) in both groups. There are no significant differences between the two groups regarding the number of affected coronary vessels.

### Circulating miRNAs expression in CAD and AMI

**Fig 1:**
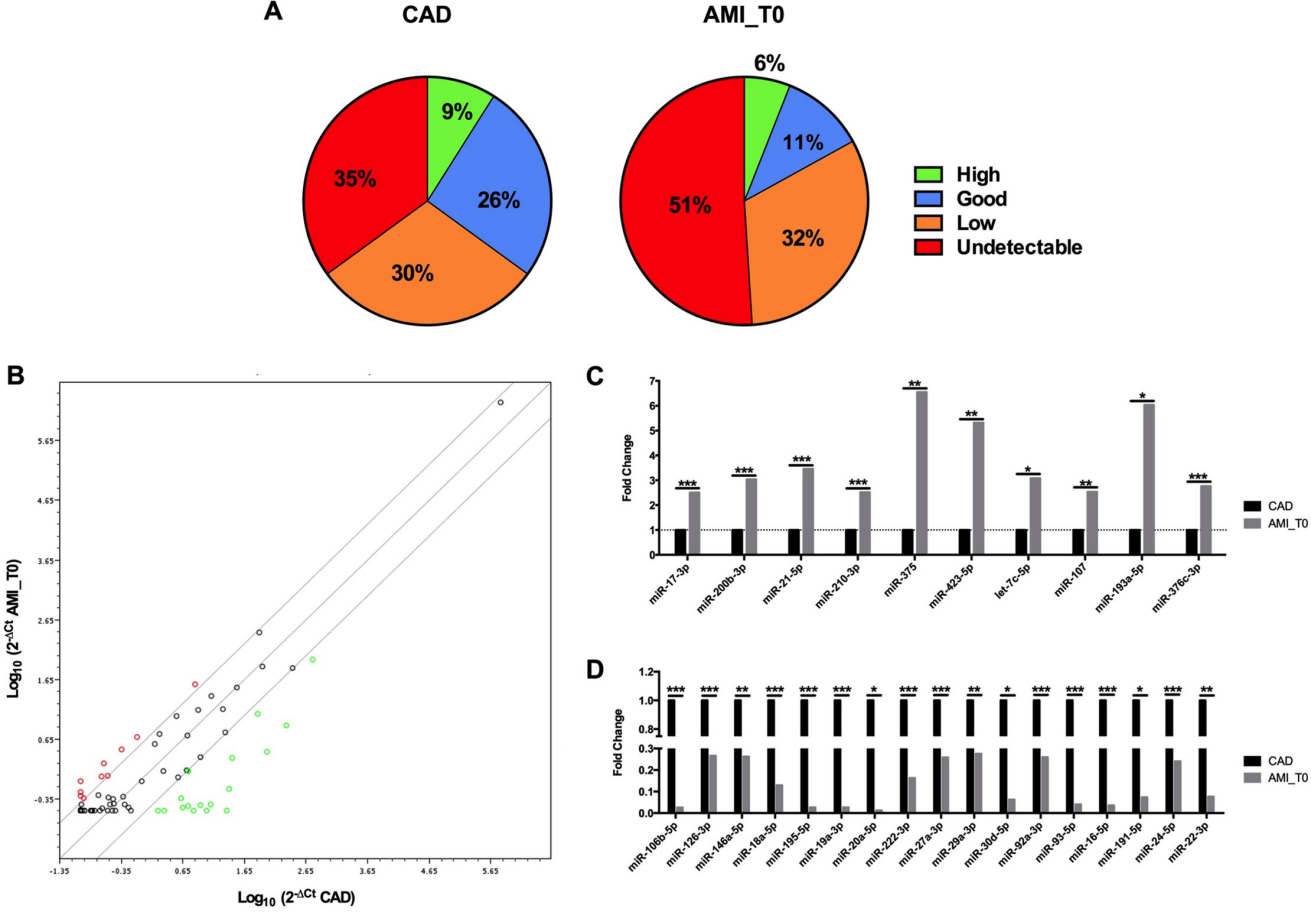
MiRNA PCR Array results. **(**A) MiRNA signature in CAD and AMI_T0 pools. MiRNAs expression is classified as high (Ct<25), good (Ct range 25–30), low (Ct range 30–35) and undetectable (Ct >35). (B) Scatter plot of 2^-ΔCT^ values showing significant differences between mature miRNA expression levels in CAD and AMI_T0. Red dots represent statistically significant up-regulated miRNAs, green dots represent statistically significant down-regulated miRNAs in AMI_T0 and black dots represent miRNAs with a comparable expression between the two plasma pools of patients. In the comparison AMI_T0 vs CAD, (C) 10 miRNAs are up-regulated and (D) 17 down-regulated (n = 3; * p≤0.05, ** p≤0.005, *** p≤0.0005).

A panel of 84 miRNAs (Human Serum & Plasma miScript miRNA PCR Array) was profiled in pooled plasma RNA of CAD (n = 5) and AMI_T0 (n = 5) patients (CAD and AMI_T0 pools). The clinical characteristics of these patients are described in S1 Table. Respectively, in CAD and AMI_T0 plasma pools, about 65% and 49% of the profiled miRNAs were detectable (Ct value <35). For CAD pool, we observed a 9% of miRNAs with a high expression level and a 26% with a good one, while a 30% of miRNAs were expressed at a low level. We detected a general lower miRNA expression level in AMI_T0 pool, with only a 6% of highly expressed miRNAs and 11% with a good expression level, while 32% of miRNAs were expressed at low levels (Fig 1A). After data normalization (GeneGlobe Data Analysis Center, www.qiagen.com), 34 circulating miRNAs were expressed with statistically significant p-value (p≤0.05) in AMI versus CAD patients (S2 Table). Among these miRNAs, 27 showed a significant two-fold up- or down-regulation (Fig 1B). Ten miRNAs resulted to be up regulated (Fig 1C) (hsa-miR-17-3p, hsa-miR-200b-3p, hsa-miR-21-5p, hsa-miR-210-3p, hsa-miR-375, hsa-miR-423-5p, hsa-let-7c-5p, hsa-miR-107, hsa-miR-193a-5p, hsa-miR-376c-3p), while 17 miRNAs were down-regulated (Fig 1D) (hsa-miR-106b-5p, hsa-miR-126-3p, hsa-miR-146a-5p, hsa-miR-18a-5p, hsa-miR-195-5p, hsa-miR-19a-3p, hsa-miR-20a-5p, hsa-miR-222-3p, hsa-miR-27a-3p, hsa-miR-29a-3p, hsa-miR-30d-5p, hsa-miR-92a-3p, hsa-miR-93-5p, miR-16-5p, miR-191-5p, miR-22-3p and miR-24-3p).

### MiR-423-5p is differentially expressed in plasma of CAD and AMI patients

**Fig 2:**
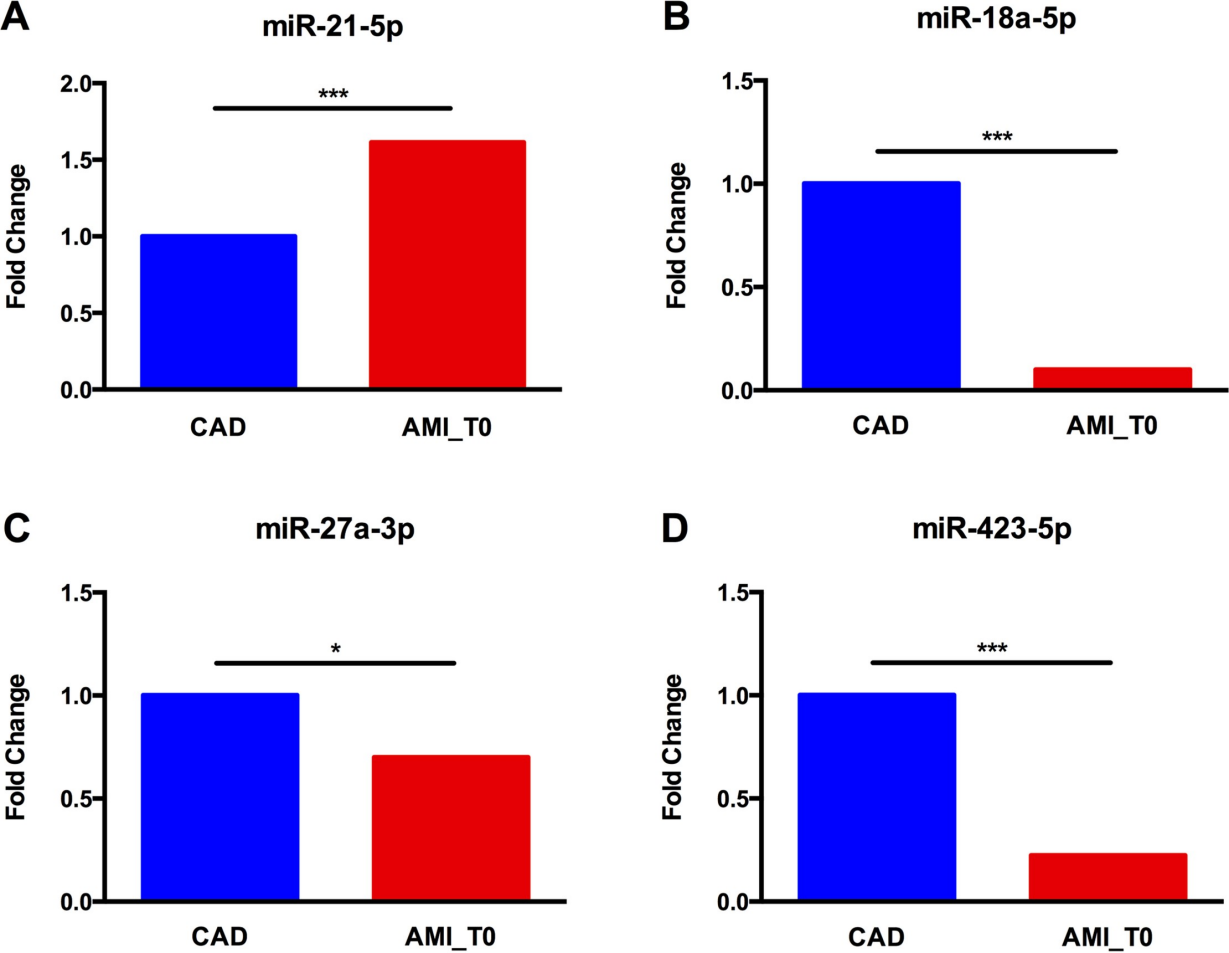
MiR-21-5p, miR-18a-5p, miR-27a-3p and miR-423-5p expression in AMI_T0 and CAD pools. Student T test, *p≤0.05, ***p≤0.0005 (n = 3).

We analyzed the 27 differentially expressed miRNAs resulted from the array experiments by qRT-PCR using specific primer assays (listed in the S1 File) on cDNA of the five samples initially used for each pool (CAD and AMI_T0). MiR-15b-5p was used for data normalization and analysis of the results. Only 4 miRNAs (miR-21-5p, FC = +1.6, p≤0.0005; miR-18a-5p, FC = -10, p≤0.0005; miR-27a-3p, FC = -1.4, p≤0.05; miR-423-5p, FC = -4.5, p≤0.0005) showed a significant expression pattern (Fig 2A–2D). Surprisingly, miR-423-5p showed an opposite expression pattern compared to the array results with a statistically significant down-regulation in AMI_T0 pool (FC = -4.5, p≤0.0005) (Fig 2D). This opposite outcome might be due to the different normalization method used for the qRT-PCRs (single housekeeping miRNA) compared to the one used for the array experiments (global normalization using the average threshold cycle of all the miRNAs present in the array plate).

**Fig 3:**
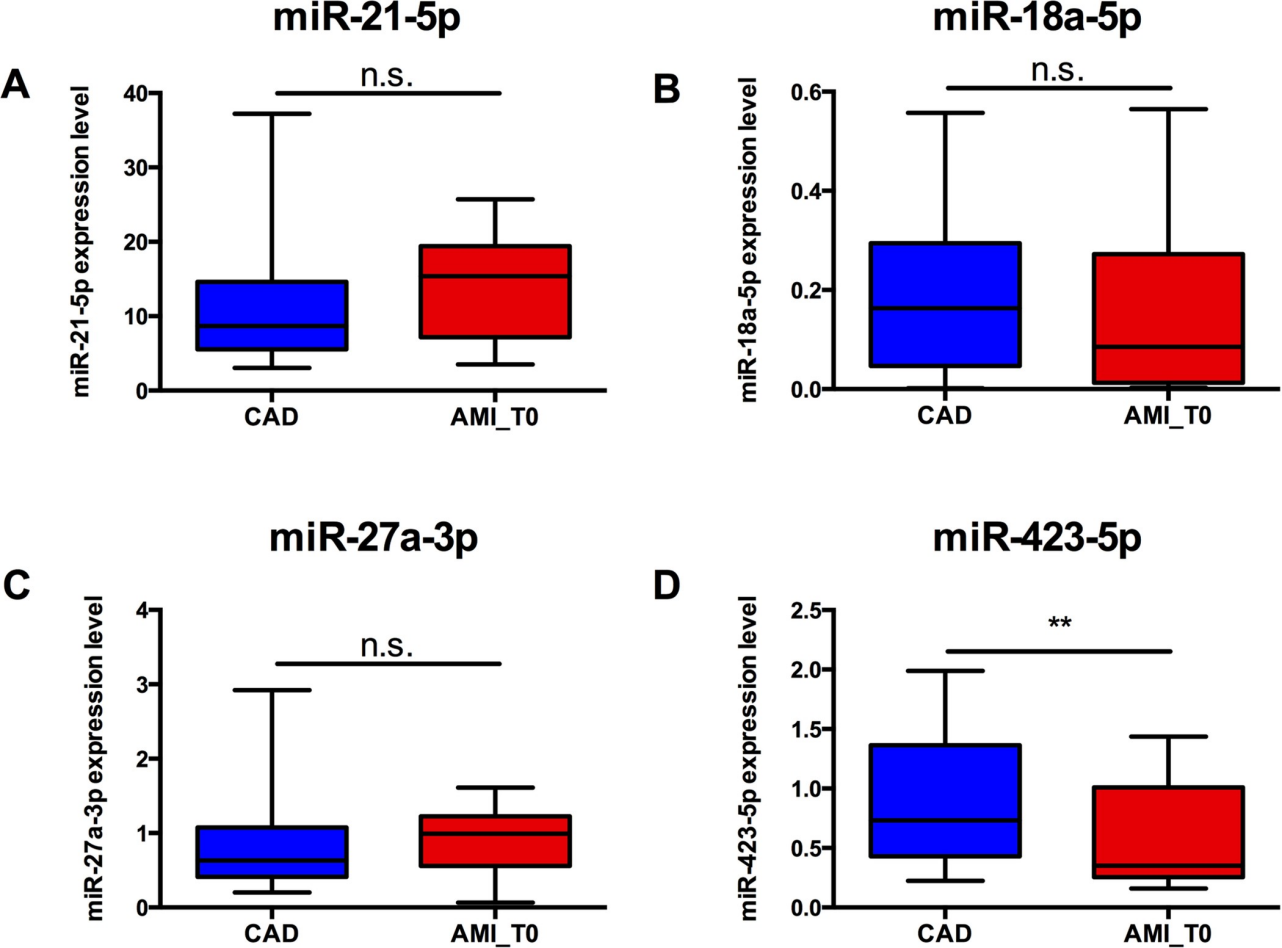
MiR-21-5p, miR-18a-5p, miR-27a-3p and miR-423-5p expression in AMI_T0 and CAD groups (n = 3). **(**A) MiR-21-5p expression in CAD and AMI_T0 groups. Mann-Whitney U = 196, n.s. **(**B) MiR-18a-5p expression in CAD and AMI_T0 groups. Mann-Whitney U = 190, n.s. **(**C) MiR-27a-3p expression in CAD and AMI_T0 groups. Mann-Whitney U = 106, n.s. **(**D) MiR-423-5p expression in CAD and AMI_T0 groups. Mann-Whitney U = 308, **p≤0.005.

**Fig 4:**
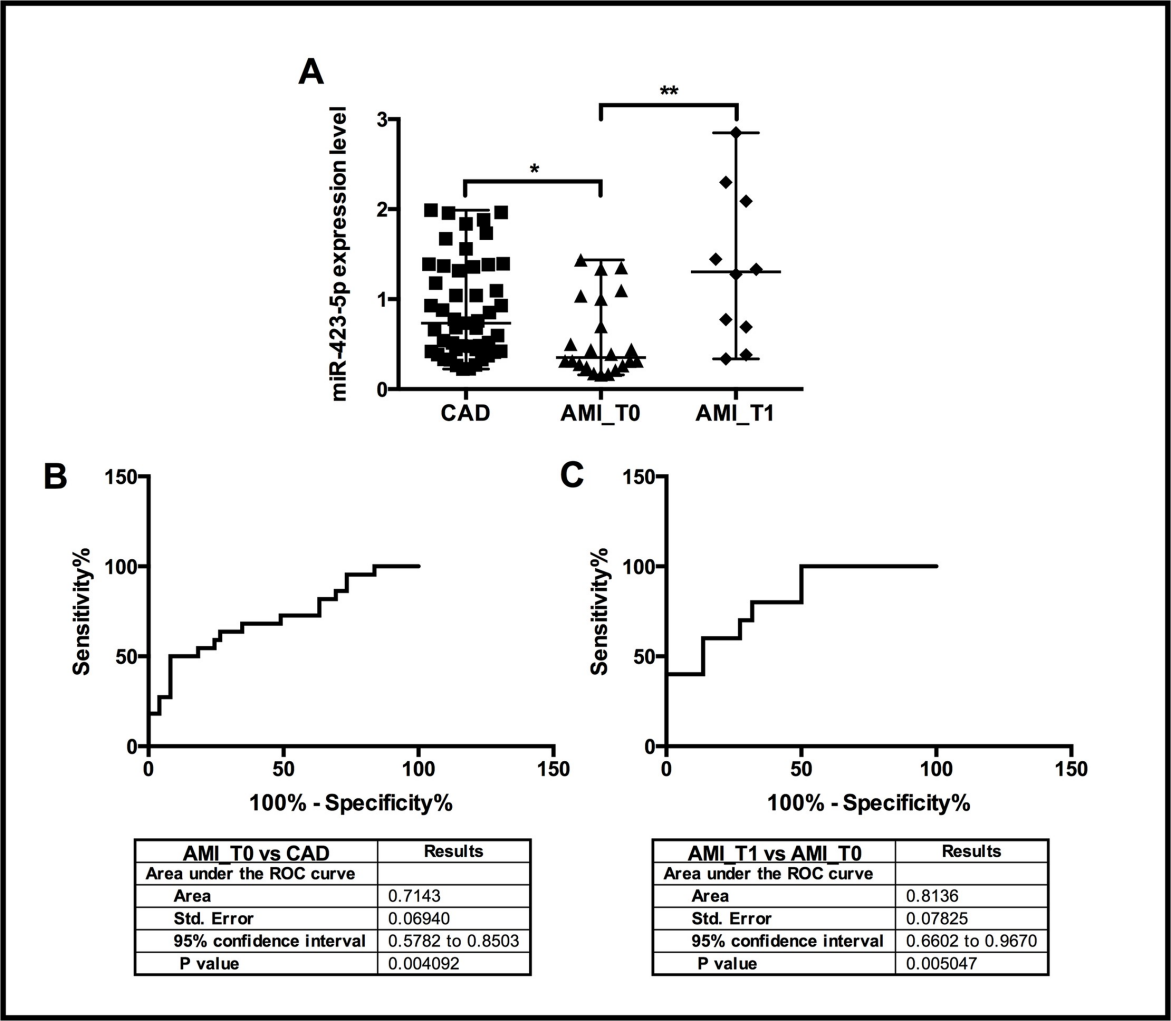
Circulating miR-423-5p expression (n = 3). (A) Circulating miR-423-5p expression in CAD, AMI_T0 and AMI_T1 groups. Kruskal-Wallis statistic 12.20, p = 0.0022; Dunn’s multiple comparisons test *p≤0.05, **p≤0.005. Discriminatory power of plasma miR-423-5p. (B) Receiver operator characteristic (ROC) curves for AMI_T0 vs CAD (set as control group). (C) Receiver operator characteristic (ROC) curves for AMI_T1 vs AMI_T0 (set as control group).

Nevertheless, we performed a qRT-PCR validation assay to analyze the expression of these 4 miRNAs on all the recruited CAD (n = 61) and AMI_T0 (n = 38) patients and interestingly, only miR-423-5p showed a significant differential expression (Fig 3A–3D). In particular, miR-423-5p resulted to be significantly down-regulated in AMI patients compared to patients with stable CAD (FC = -2, p≤0.05) (Figs 3D and 4A). In order to evaluate if the expression of plasma miR-423-5p might change with clinical features, we started the recruitment of AMI patients at 6 months post-AMI (AMI_T1 group, n = 11). Interestingly, miR-423-5p resulted to be significantly up-regulated (FC = +2, p≤0.005) in unstable patients 6 months post-AMI (AMI_T1) in comparison with AMI_T0 patients while its expression resulted to be unchanged compared to stable CAD patients (Fig 4A). To investigate the potential of miR-423-5p as circulating biomarker useful for the discrimination of patients with stable and unstable CAD, we performed ROC analyses. MiR-423-5p showed the best discriminatory power (AUC = 0.814; 95% confidence interval 0.6602–0.9670; p-value ≤0.005) in the comparison between patients with unstable CAD at 6 months post-AMI (AMI_T1) and the same patients during the acute event (AMI_T0). (Fig 4C) This result suggests that miR-423-5p expression level might be associated to an improved stability of the disease in patients with CAD.

### Evaluation of miR-423-5p in PBMCs and ROC curve

**Fig 5:**
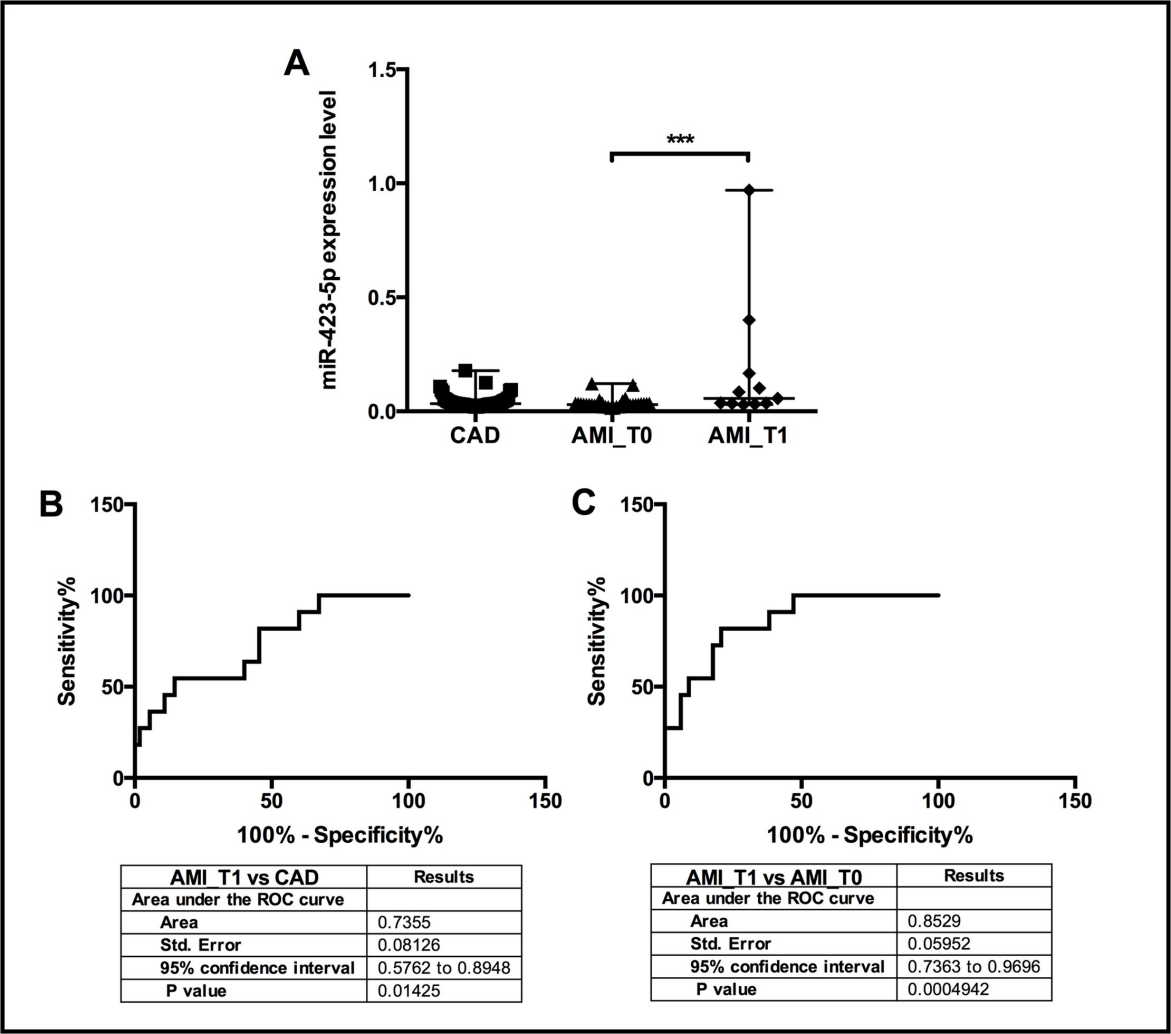
MiR-423-5p expression in PBMCs (n = 3). (A) MiR-425-5p expression in PBMCs of CAD, AMI_T0 and AMI_T1 patients. Kruskal-Wallis statistic 11.93, p≤0.005. Discriminatory power of miR-423-5p expression in PBMCs. (B) Receiver operator characteristic (ROC) curves for AMI_T1 vs CAD (set as control group). (C) Receiver operator characteristic (ROC) curves for AMI_T1 vs AMI_T0 (set as control group).

Since it is known that gene expression in PBMCs might be a mirror of the gene expression pattern of a specific tissue thus reflecting its pathophysiological status, we also evaluated the expression pattern of miR-423-5p in PBMCs of all the patients recruited in the study (Fig 5). According to plasma results, cellular miR-423-5p expression resulted to be significantly up-regulated 6 months post-AMI (AMI_T1) respect to 24h after AMI (p≤0.0005). No differences were observed for its expression compared to patients with stable CAD (Fig 5A).

To investigate the discriminatory power of miR-423-5p in PBMCs, we conducted a ROC analysis. Also, in PBMCs the highest AUC value (AUC of 0.8529; 95% confidence interval 0.7363 to 0.9696; p≤0.0005) was found when comparing patients with unstable CAD after six months from AMI (AMI_T1) with the same patients during the acute event (AMI_T0) (Fig 5C).

### Correlation analysis of miRNA-423-5p expression in plasma and PBMCs

**Fig 6:**
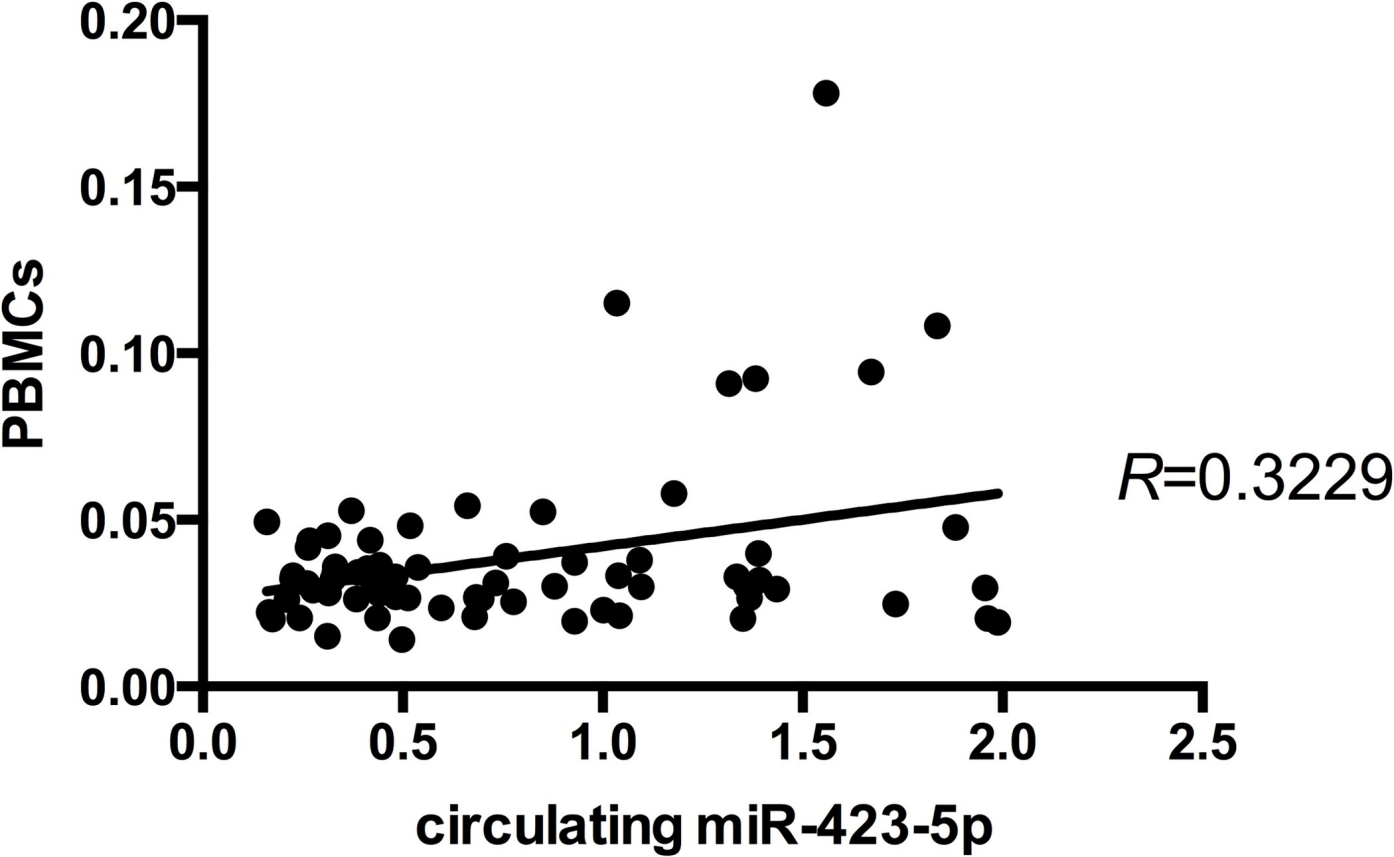
Pearson correlation. Pearson correlation scatter plot of plasma and PBMCs miR-423-5p (R = 0.3229; p = 0.006).

In order to assess if miR-423-5p expression in plasma and PBMCs are correlated, we performed a Pearson correlation analysis considering CAD and AMI_T0 patients as one group. The analysis showed a significant positive correlation between miR-423-5p plasma and PBMCs levels (R = 0.3229; p **=** 0.006) (Fig 6).

## Discussion

Cardiovascular disease (CVD) is a recognized age-dependent condition whose incidence is expected to rise due to the aging of population [21,22]. In Europe, more than the 60% of all cardiovascular deaths occurs in people aged 75 years or older [23]. It is well known that CVD and CAD risk depends on modifiable (i.e. environmental aspects) and non-modifiable (i.e. genetic aspects) risk factors [5]. Moreover, during the life course, a variety of environmental stimuli is able to induce changes of histone/DNA complexes or alter the expression of non-coding RNAs, such as miRNAs. Concerning atherosclerosis and CAD, wide epigenetic changes occur in endothelial cells, vascular smooth muscle cells and macrophages, influencing the expression of many genes involved in the alteration of a number of pathways leading to the development of the atherosclerotic plaque [24]. Undoubtedly, for patient stratification and prediction of cardiovascular disease risk it is crucial to create algorithms that coupled epigenetic and genetic background with most classical risk factors [25,26].

This pilot-study is aimed to compare the differences in circulating miRNAs’ expression between patients with stable CAD and patients with AMI; therefore, we did not consider healthy subjects. After a profiling of the most common circulating miRNAs in these patients, we focused our attention on the differential expression of miR-423-5p suggesting its potential use as an epigenetic biomarker for risk stratification of CAD patients.

In AMI patients, plasma levels of miR-423-5p resulted to be down-regulated within 24 hours from the acute event (AMI_T0 patients) and increased at 6 months post-AMI (AMI_T1 patients). Moreover, the evaluation of miR-423-5p expression pattern in PBMCs strengthened this finding showing an increased expression of miR-423-5p in AMI_T1 patients. These results are supported by the ROC analyses that showed a good discriminatory potential of miR-423-5p (both in plasma and PBMCs) for the identification of patients with stable CAD from patients with unstable disease (AMI patients). Considering our CAD and AMI patients as one group regardless their medical condition, we performed a Pearson correlation based-metric that showed a significant correlation between miR-423-5p expression level in plasma and PBMCs (R = 0.3229; p = 0.006), suggesting the possible comparability of miR-423-5p expression in circulation and PBMCs.

MiR-423-5p is located within the first intron of nuclear speckle splicing regulatory protein 1 gene (*NSRP1*) on chromosome 17 [27]. MiR-423-5p has been investigated in case-control studies, as possible biomarker in heart failure (HF) [28,29] and coronary artery disease [30]. In HF, plasmatic miR-423-5p levels positively correlated with peripheral N-terminal pro-BNP values corroborating the cardiac specificity of miR-423-5p [31]. A role for miR-423-5p has also been speculated in Lupus nephritis (LN), a kidney disorder resulting from the autoimmune inflammatory disease systemic lupus erythematosus (SLE) [31]. In LN patients, miR-423-5p targets *TNIP2*, a negative regulator of NF-kB, suggesting the involvement of miR-423-5p in the TNIP2-NF-kB axis [28]. These findings raise the idea of investigating a possible involvement of miR-423-5p in coronary artery disease inflammatory background.

Regarding coronary artery disease, Nabialek et al [30] analyzed the expression of miR-423-5p in patients with stable CAD and patients with AMI in comparison with healthy subjects. They observed a statistically significant up-regulation of miR-423-5p in AMI patients before pPCI compared to controls, while no significant differences in miR-423-5p expression were detected at the follow-ups post pPCI. On this basis, they indicate miR-423-5p as an early marker of myocardial necrosis. Interestingly, our data indicating a differential expression of miR-423-5p, are obtained from the comparison of stable and unstable CAD patients after PCI, so pointing to the role of miR-423-5p as a potent epi-biomarker for the evaluation of AMI risk.

Moreover, a functional role of miR-423-5p in cardiomyocytes apoptosis is suggested by studies in mice demonstrating that m-O-GlcNAc transferase (*OGT*) gene is a target of m-miR-423-5p [32]. OGT, which adds the simple sugar O-GlcNAc (β-O-linked N-acetylglucosamine) to Serine or Threonine residues of target proteins, is required for cell division and embryogenesis [33]. In murine cardiomyocytes, the expression of m-miR-423-5p led to the inhibition of both m-OGT expression and phosphorylation of *AMPK*, an m-OGT downstream target. Consequently, expression levels of the pro-apoptotic proteins p53 and caspase-3, which are downstream targets of *AMPK*, were increased by m-miR-423-5p. Accordingly, the apoptotic rate of cardiomyocytes was increased after transfection with miR-423-5p [32]. Moreover, the ablation of OGT from cardiomyocytes is involved in the induction of heart failure; indeed, OGT is an important part of the endogenous compensatory response to infarct-induced heart failure [34]. Noteworthy, apoptotic death of cardiomyocytes in the border zone of myocardial infarcted area aggravates cardiac dysfunction causing heart failure and mortality [35]. Additionally, i*n silico* target predictions and literature data suggest that miR-423-5p might have a potential role in the regulation of transcription factors involved in several cellular processes, such as proliferation and differentiation [36–37]. Among its target genes, experimental evidences showed *PA2G4* gene as a direct target of miR-423-5p [38]. *PA2G4* encodes a cell-cycle protein able to interact with DNA, RNA and proteins and involved in the regulation of cell proliferation, differentiation and survival. In particular, *PA2G4* induce cell cycle arrest in G2/M phase [39] through the transcriptional repression of E2F1-regulated genes [40]. Recently, there are growing evidence that PA2G4 protein family can exert anti-apoptotic effects in cardiomyocytes [41]. Overall, these data indicate a functional pro-apoptotic role of miR-423-5p in cardiomyocytes. Accordingly, miR-423-5p down-regulation in plasma and PBMCs in the first 24 hours from the AMI event, might be considered as a “cardiomyocytes mirror” reflecting a “physiological” mechanism of protection, aimed to reduce cardiomyocytes apoptosis (through enhanced expression of anti-apoptotic target genes) and to promote cardiac repair during early stages of AMI.

## Conclusions

This pilot-study has been carried out by comparing unstable CAD patients during an AMI event to stable CAD patients, not considering healthy control subjects as in previous studies [12,16,42–44]. We showed that miR-423-5p expression level demonstrated a good discriminatory ability to separate stable elder CAD patients from patients with AMI. This pilot-study confirms the need of deeper investigations on miR-423-5p as a new epigenetic biomarker with diagnostic and, hopefully in future, prognostic value.

Although this research was carefully prepared, we are aware of some limitations and shortcomings. First, the sample size analyzed in this pilot-study is low. Second, previously reported circulating miRNAs in acute myocardial infarction (i.e., miR-133, 208a, miR-1 and miR-499 [10]), did not result to be significant expressed among our patients. The reason for this result could be addressed to the time frame in which the samples were collected and to the clinical characteristics of the patients.

## Supporting information

S1 Table Clinical characteristics of CAD and AMI patients selected for Human Serum & Plasma miScript miRNA PCR Array experiments. Pooled plasma RNA samples obtained from CAD (n = 5) and AMI_T0 (n = 5) patients, matched for age and clinical characteristics according to their medical condition.
(DOCX)

S2 Table Human Serum & Plasma miScript miRNA PCR Array raw data (2^-Avg.(Δ Ct)^). Data shown are the mean of three independent experiments.
(DOCX)

S1 File In the table are listed the 27 miRNAs resulted differentially expressed from the array experiments by qRT-PCR and then analyzed using specific primer assays. (DOCX)

S1 Fig Expression analysis of miR-15-5p in CAD, AMI_T0 and AMI_T1 groups. Average threshold cycle (Ct) of miR-15-5p in CAD, AMI_T0 and AMI_T1 groups. Data analysis was performed using the comparative Ct method quantification (2^-ΔCt^ method).
(TIF)
