# Supplementary material for: MiR-423 is differentially expressed in patients with stable and unstable coronary artery disease: A pilot study
Source: PLoS One. 2019 May 6;14(5):e0216363. doi: 10.1371/journal.pone.0216363 (PMC6502321; doi:10.1371/journal.pone.0216363)
Supplement: S1 Table — Pooled plasma RNA samples obtained from CAD (n = 5) and AMI_T0 (n = 5) patients, matched for age and clinical characteristics according to their medical condition. (DOCX) [file pone.0216363.s001.docx]

S1 Table.

|  | **POOL CAD** | **POOL AMI_T0** | **p-value** |
| --- | --- | --- | --- |
| **Age (years)** | 70.8±3.4 | 71.2±11 | n.s. |
|  |  |  |  |
| **Family history (%)** | 60 | 40 | n.s. |
| **Hypertension (%)** | 100 | 100 | n.s. |
| **Diabetes (%)** | 40 | 60 | n.s. |
| **Dyslipidemia (%)** | 100 | 100 | n.s. |
| **Present smokers (%)** | 100 | 100 | n.s. |
| **Number of affected vessels** |  |  |  |
| **1 vessel disease (%)** | 60 | 40 | n.s. |
| **2 vessel disease (%)** | 20 | 60 | n.s. |
| **3 vessel disease (%)** | 20 | 0 | n.s. |
| **Type of affected vessel** |  |  |  |
| **LAD^a^ (%)** | 80 | 80 | n.s. |
| **CFX^b^ (%)** | 40 | 20 | n.s. |
| **RCA^c^ (%)** | 40 | 40 | n.s. |
| **Troponin I (ng/mL)** | - | 144±96 |  |

Continuous data are expressed as mean ± SD; categorical data are expressed as percentage. Student T test was used to assess significance.

^a^LAD, left descending artery.

^b^CFX, circumflex coronary artery.

_c_RCA, right coronary artery.
