# Supplementary material for: MiR-423 is differentially expressed in patients with stable and unstable coronary artery disease: A pilot study
Source: PLoS One. 2019 May 6;14(5):e0216363. doi: 10.1371/journal.pone.0216363 (PMC6502321; doi:10.1371/journal.pone.0216363)
Supplement: S2 Table — Data shown are the mean of three independent experiments. (DOCX) [file pone.0216363.s002.docx]

S2 Table.

|  |  | **2^-Avg.( ΔCt)^** | | **Fold Change (comparing to CAD pool)** | **p-value** |
| --- | --- | --- | --- | --- | --- |
| **Position** | **Mature ID** | **CAD pool** | **AMI_T0 pool** |  |  |
| A01 | hsa-let-7a-5p | Undetermined | Undetermined |  |  |
| A02 | hsa-miR-1-3p | 0.227656 | 1.774028 | 7.7926 | 0.111898 |
| A03 | hsa-miR-100-5p | 1.87202 | 5.411502 | 2.8907 | 0.061823 |
| A04 | hsa-miR-106b-5p | 10.921477 | 0.281856 | -38.76 | 0.000002 |
| A05 | hsa-miR-10b-5p | 0.266757 | 1.093936 | 4.1009 | 0.174087 |
| A06 | hsa-miR-122-5p | 640836.1529 | 1918475.6 | 2.9937 | 0.363076 |
| A07 | hsa-miR-124-3p | 0.325357 | 0.440291 | 1.3533 | 0.393593 |
| A08 | hsa-miR-125b-5p | 0.948747 | 0.883027 | 0.9307 | 0.342127 |
| A09 | hsa-miR-126-3p | 21.865676 | 5.818023 | -3.758 | 0.000001 |
| A10 | hsa-miR-133a-3p | Undetermined | Undetermined |  |  |
| A11 | hsa-miR-133b | Undetermined | Undetermined |  |  |
| A12 | hsa-miR-134-5p | Undetermined | Undetermined |  |  |
| B01 | hsa-miR-141-3p | Undetermined | Undetermined |  |  |
| B02 | hsa-miR-143-3p | 0.149298 | 0.281856 | 1.8879 | 0.038292 |
| B03 | hsa-miR-146a-5p | 5.100349 | 1.339346 | -3.808 | 0.002192 |
| B04 | hsa-miR-150-5p | Undetermined | Undetermined |  |  |
| B05 | hsa-miR-155-5p | 0.097368 | 0.873288 | 8.9689 | 0.055189 |
| B06 | hsa-miR-17-5p | 12.960918 | 23.670676 | 1.8263 | 0.373106 |
| B07 | hsa-miR-17-3p | 0.112547 | 0.281856 | 2.5043 | 0.000429 |
| B08 | hsa-miR-18a-5p | 2.174368 | 0.281856 | -7.716 | 0.000121 |
| B09 | hsa-miR-192-5p | 2.142204 | 1.28123 | -1.672 | 0.510430 |
| B10 | hsa-miR-195-5p | 102.579314 | 2.704813 | -37.879 | 0.000001 |
| B11 | hsa-miR-196a-5p | 0.264547 | 0.281856 | 1.0654 | 0.716724 |
| B12 | hsa-miR-19a-3p | 25.073579 | 0.65931 | -38.023 | 0.000002 |
| C01 | hsa-miR-19b-3p | Undetermined | Undetermined |  |  |
| C02 | hsa-miR-200a-3p | Undetermined | Undetermined |  |  |
| C03 | hsa-miR-200b-3p | 0.097368 | 0.295732 | 3.0373 | 0.000503 |
| C04 | hsa-miR-200c-3p | 0.190333 | 0.51028 | 2.681 | 0.353317 |
| C05 | hsa-miR-203a-3p | Undetermined | Undetermined |  |  |
| C06 | hsa-miR-205-5p | 0.163413 | 0.281856 | 1.7248 | 0.563875 |
| C07 | hsa-miR-208a-3p | Undetermined | Undetermined |  |  |
| C08 | hsa-miR-20a-5p | 23.120425 | 0.281856 | -81.967 | 0.000014 |
| C09 | hsa-miR-21-5p | 77.848429 | 269.385612 | 3.4604 | 0.000089 |
| C10 | hsa-miR-210-3p | 0.111899 | 0.281856 | 2.5189 | 0.000350 |
| C11 | hsa-miR-214-3p | 0.141784 | 0.281856 | 1.9879 | 0.009528 |
| C12 | hsa-miR-215-5p | Undetermined | Undetermined |  |  |
| D01 | hsa-miR-221-3p | 0.31373 | 0.281856 | -1.1131 | 0.567636 |
| D02 | hsa-miR-222-3p | 1.760025 | 0.287048 | -6.1312 | 0.000493 |
| D03 | hsa-miR-223-3p | 86.228687 | 73.492697 | -1.1733 | 0.041631 |
| D04 | hsa-miR-224-5p | 0.360631 | 0.281856 | -1.2794 | 0.252972 |
| D05 | hsa-miR-23a-3p | 33.628115 | 32.234395 | -1.0432 | 0.654065 |
| D06 | hsa-miR-25-3p | Undetermined | Undetermined |  |  |
| D07 | hsa-miR-27a-3p | 8.494894 | 2.194708 | -3.87 | 0.000959 |
| D08 | hsa-miR-296-5p | 0.097368 | 0.579426 | 5.9509 | 0.087420 |
| D09 | hsa-miR-29a-3p | 3.755737 | 1.036004 | -3.6258 | 0.017175 |
| D10 | hsa-miR-30d-5p | 5.408003 | 0.338532 | -15.974 | 0.000036 |
| D11 | hsa-miR-34a-5p | 0.145366 | 0.281856 | 1.9389 | 0.046262 |
| D12 | hsa-miR-375 | 0.455365 | 2.982514 | 6.5497 | 0.000964 |
| E01 | hsa-miR-423-5p | 6.945584 | 36.950679 | 5.32 | 0.000610 |
| E02 | hsa-miR-499a-5p | Undetermined | Undetermined |  |  |
| E03 | hsa-miR-574-3p | 0.277988 | 0.466634 | 1.6786 | 0.519539 |
| E04 | hsa-miR-885-5p | 0.472569 | 0.485439 | 1.0272 | 0.667230 |
| E05 | hsa-miR-9-5p | Undetermined | Undetermined |  |  |
| E06 | hsa-miR-92a-3p | 268.838512 | 69.842291 | -3.8491 | 0.000013 |
| E07 | hsa-miR-93-5p | 8.700486 | 0.352256 | -24.691 | 0.000033 |
| E08 | hsa-let-7c-5p | 3.517611 | 10.838018 | 3.0811 | 0.029388 |
| E09 | hsa-miR-107 | 0.111177 | 0.281856 | 2.5352 | 0.000919 |
| E10 | hsa-miR-10a-5p | 0.211994 | 1.061077 | 5.0052 | 0.213784 |
| E11 | hsa-miR-128-3p | 0.575982 | 0.322126 | -1.7879 | 0.011318 |
| E12 | hsa-miR-130b-3p | 6.699698 | 0.281856 | -23.753 | 0.160099 |
| F01 | hsa-miR-145-5p | 0.291405 | 0.36024 | 1.2362 | 0.620567 |
| F02 | hsa-miR-148a-3p | 5.366926 | 1.282562 | -4.1841 | 0.091756 |
| F03 | hsa-miR-15a-5p | 0.198209 | 0.281856 | 1.422 | 0.038993 |
| F04 | hsa-miR-184 | Undetermined | Undetermined |  |  |
| F05 | hsa-miR-193a-5p | 0.801401 | 4.83672 | 6.0353 | 0.010989 |
| F06 | hsa-miR-204-5p | Undetermined | Undetermined |  |  |
| F07 | hsa-miR-206 | Undetermined | Undetermined |  |  |
| F08 | hsa-miR-211-5p | Undetermined | Undetermined |  |  |
| F09 | hsa-miR-26b-5p | 7.92327 | 13.571695 | 1.7129 | 0.145246 |
| F10 | hsa-miR-30e-5p | Undetermined | Undetermined |  |  |
| F11 | hsa-miR-372-3p | Undetermined | Undetermined |  |  |
| F12 | hsa-miR-373-3p | Undetermined | Undetermined |  |  |
| G01 | hsa-miR-374a-5p | 0.331733 | 0.367215 | 1.107 | 0.618776 |
| G02 | hsa-miR-376c-3p | 0.101973 | 0.281856 | 2.764 | 0.000016 |
| G03 | hsa-miR-7-5p | 1.556274 | 3.702575 | 2.3791 | 0.267347 |
| G04 | hsa-miR-96-5p | Undetermined | Undetermined |  |  |
| G05 | hsa-miR-103a-3p | 0.505084 | 0.364089 | -1.3873 | 0.461655 |
| G06 | hsa-miR-15b-5p | 20.106583 | 14.025973 | -1.4335 | 0.005070 |
| G07 | hsa-miR-16-5p | 213.722652 | 7.639769 | -28.011 | 0.000001 |
| G08 | hsa-miR-191-5p | 4.368373 | 0.322349 | -13.55 | 0.000578 |
| G09 | hsa-miR-22-3p | 28.494193 | 2.16975 | -13.141 | 0.001322 |
| G10 | hsa-miR-24-3p | 5.339098 | 1.284342 | -4.1563 | 0.000240 |
| G11 | hsa-miR-26a-5p | 5.28937 | 5.182072 | -1.0207 | 0.826095 |
| G12 | hsa-miR-31-5p | Undetermined | Undetermined |  |  |
| H01 | cel-miR-39-3p | 562.650804 | 94.32243 | NA |  |
| H02 | cel-miR-39-3p | 73.776836 | 11.897025 | NA |  |
| H03 | SNORD61 | Undetermined | Undetermined | NA |  |
| H04 | SNORD68 | Undetermined | Undetermined | NA |  |
| H05 | SNORD72 | Undetermined | Undetermined | NA |  |
| H06 | SNORD95 | 0.62942 | Undetermined | NA |  |
| H07 | SNORD96A | 0.221073 | 0.30602 | NA |  |
| H08 | RNU6-6P | Undetermined | Undetermined | NA |  |
| H09 | miRTC | 424.688926 | 1229.363339 | NA |  |
| H10 | miRTC | 440.173764 | 1274.187893 | NA |  |
| H11 | PPC | 13493.05442 | 44055.33101 | NA |  |
| H12 | PPC | 14704.08456 | 49188.34811 | NA |  |
