# Supplementary material for: MiR-423 is differentially expressed in patients with stable and unstable coronary artery disease: A pilot study
Source: PLoS One. 2019 May 6;14(5):e0216363. doi: 10.1371/journal.pone.0216363 (PMC6502321; doi:10.1371/journal.pone.0216363)
Supplement: S1 File — (DOCX) [file pone.0216363.s003.docx]

**S1 File.**

| **miRNA** | **QIAGEN catalog number** |
| --- | --- |
| hsa-miR-17-3p | MS00006524 |
| hsa-miR-200b-3p | MS00009016 |
| hsa-miR-21-5p | MS00009079 |
| hsa-miR-210-3p | MS00003801 |
| hsa-miR-375 | MS00031829 |
| hsa-miR-423-5p | MS00009681 |
| hsa-let-7c-5p | MS00003129 |
| hsa-miR-107 | MS00031255 |
| hsa-miR-193a-5p | MS00008932 |
| hsa-miR-376c-3p | MS00004046 |
| hsa-miR-106b-5p | MS00003402 |
| hsa-miR-126-3p | MS00003430 |
| hsa-miR-146a-5p | MS00003535 |
| hsa-miR-18a-5p | MS00031514 |
| hsa-miR-195-5p | MS00003703 |
| hsa-miR-19a-3p | MS00003192 |
| hsa-miR-20a-5p | MS00003192 |
| hsa-miR-222-3p | MS00007609 |
| hsa-miR-27a-3p | MS00003241 |
| hsa-miR-29a-3p | MS00003262 |
| hsa-miR-30d-5p | MS00009387 |
| hsa-miR-92a-3p | MS00006594 |
| hsa-miR-93-5p | MS00003346 |
| hsa-miR-16-5p | MS00031493 |
| hsa-miR-191-5p | MS00003682 |
| hsa-miR-22-3p | MS00003220 |
| hsa-miR-24-3p | MS00006552 |
| hsa-miR-15b-5p | MS00008792 |
