# Supplementary figures and images for: MiR-423 is differentially expressed in patients with stable and unstable coronary artery disease: A pilot study
Source: PLoS One. 2019 May 6;14(5):e0216363. doi: 10.1371/journal.pone.0216363 (PMC6502321; doi:10.1371/journal.pone.0216363)

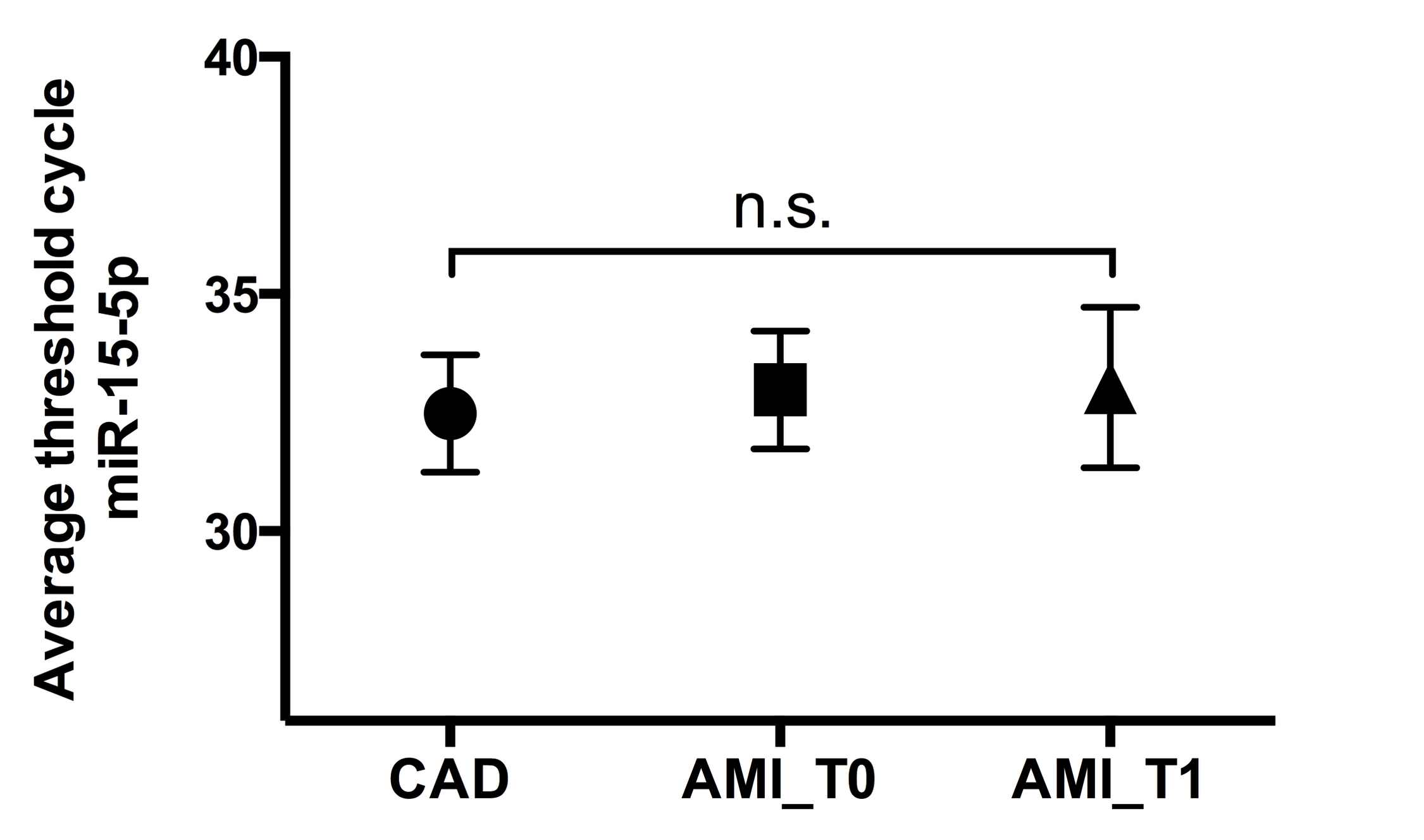

Supplement: S1 Fig — Average threshold cycle (Ct) of miR-15-5p in CAD, AMI_T0 and AMI_T1 groups. Data analysis was performed using the comparative Ct method quantification (2-ΔCt method). (TIF) [file pone.0216363.s004.tif]
